# Dr. Thomas W. Evans

**Published:** 1897-12

**Authors:** 


### BIOGRAPHICAL.

### Dr. Thomas W. Evans.

Dr. Thomas W. Evans the noted American dentist of Paris, France, died suddenly at his home on the evening of November 15th. Dr. Evans was born in Philadelphia about seventy-five years ago. After preparing himself for the practice of dentistry in his native city, he in 1846 went to Paris and there at once began practice and attained a remarkable success. He had a career unparalled in the history of the profession. Dr. Evans was exceedingly fortunate in the attainment of desirable position and standing, which gave him very great opportunities for lucrative practice and for bringing dentistry and its resources to the notice of many of the most prominent people of European countries.

Dr. Evans did two things while in Paris, that especially drew attention to him and to what he did; the one was, securing the acquaintance and friendship of so many prominent people in the countries of Europe; and the second was, the attainment of an unusual amount of money, at least for a dentist. The latter he did however, largely outside of dental practice. Dr. Evan's wealth has been estimated (by newspaper reporters,) at from three to fifty million dollars; doubtless he left a large estate.

In the statements that have been made in regard to Dr. Evans, several inaccuracies have crept in. It has been said that he was the first American dentist to establish a practice in Paris. Dr. C. Starr Brewster, M, D., D. D. S., went to Paris before 1842 and established a very successful practice, not only in Paris, but was called to other European countries, and was made by the Emperor of Russia, Knight of the Order St. Stanislas, and Dentiste Honorare of the Russian Court.

The statement is also made that Dr. Evans was the first person to introduce gold filling for teeth. These are only examples of statements made by persons ignorant of, or reckless in regard to the facts of history. Dr. Evans certainly can not be held responsible for such statements, and they are calculated to do him injury rather than credit.

In August last, Dr. Evans lost his wife, which was a great and severe blow to him, aud from which he did not fully recover. He brought the remains of his life companion to the city of her nativity, Philadelphia, for interment.

After a time of rest he visited a number of the larger cities of this country, but he failed to receive the recuperation for which he and his friends hoped, and he returned to his Paris home early in the latter part of October, where his physical condition became worse and he died as above stated. Dr. Evans made for himself a name and a reputation that is recognized wherever dentistry is known throughout the world, and nowhere better than in his native land.

